# Allelic and Genotypic Analysis of LncRNA ANRIL rs4977574 A/G Mutations in Oral Squamous Cell Carcinoma Patients: Insights into Tumor Characteristics and Genotypic Correlations

**DOI:** 10.1155/2023/7738719

**Published:** 2023-10-04

**Authors:** Mohammad Amin Amiri, Delara Amiri, Mohammad Javad Mokhtari, Fatemeh Lavaee, Mohammad Javad Fattahi, Abbas Ghaderi, Bijan Khademi

**Affiliations:** ^1^Student Research Committee, Shiraz University of Medical Sciences, Shiraz, Iran; ^2^Department of Biology, Zarghan Branch, Islamic Azad University, Zarghan, Iran; ^3^Oral and Dental Disease Research Center, Shiraz University of Medical Sciences, Shiraz, Iran; ^4^Shiraz Institute of Cancer Research, School of Medicine, Shiraz University of Medical Sciences, Shiraz, Iran; ^5^Department of Otorhinolaryngology, Khalili Hospital, Faculty of Medicine, Shiraz University of Medical Sciences, Shiraz, Iran

## Abstract

**Aim:**

Long noncoding RNAs (lncRNA) ANRIL and its genetic polymorphisms are shown to be associated with the risk of several cancers. However, the single nucleotide polymorphisms (SNPs) of lncRNA ANRIL are not thoroughly assessed in oral squamous cell carcinoma (OSCC) which is the most prevalent cancer in the head and neck area. Thus, this study aimed to assess the association of SNP of lncRNA ANRIL rs4977574 in patients with OSCC.

**Methods and Materials:**

106 blood samples from the patients with OSCC were obtained with a gender- and age-matched control group to evaluate the SNP of rs4977574 of lncRNA ANRIL. The DNA was extracted using the salt-out technique and DNA genotyping was undertaken using specific primer pairs in the tetra-primer ARMS-PCR technique. Eventually, the frequency of wild-type (A) and the mutated allele (G), as well as the genotypes were estimated between the groups of patients with OSCC and healthy individuals.

**Results:**

The results of our study indicated no statistically significant difference in the frequency of rs4977574 A/G of lncRNA ANRIL among the patients with OSCC and healthy individuals (*p* > 0.05). Likewise, no significant difference was found in the genotypes' frequencies (*p* > 0.05). Nevertheless, the marked association of GG with smaller tumor size and the high level of differentiation of OSCC cells in the presence of AA or AG genotypes were interesting outcomes of this study (*p* < 0.05). Similarly, all the genotypes AA, AG, and GG were correlated with the site of the occurrence of OSCC. Furthermore, the association of the genotypes with the lymph node metastasis and the tumors stage was not found to be significant (*p* > 0.05).

**Conclusions:**

The results of our study indicate that rs4977574 A/G and its genotypes do not have any direct correlation with the presence of OSCC; however, its association with the smaller tumor size and the level of the cancer cells differentiation could imply its possible indirect role.

## 1. Introduction

Oral squamous cell carcinoma (OSCC), the most common cancer in the oral cavity, constitutes 90% of the oral malignant lesions [1]. One of the major concerns in patients with OSCC could be their 5-year survival rate which is estimated as 50%, while the late diagnosis of this cancer could further worsen the prognosis and the course of the cancer progression [2]. This malignant oral cancer is associated with multiple genetic changes, especially single nucleotide polymorphisms (SNPs) resulting in genome instability, and hence a disturbance in normal cellular mechanisms [3–6]. These SNPs could be potential indicators for the risk of oral cancer which entail further assessments concerning their effectiveness in this regard.

In accordance with the current evidence, several potential biomarkers are suggested as possible indicators of the development and prognosis of OSCC [7]. One of the possible biomarkers with interesting results in this regard is long noncoding RNAs (lncRNAs) [8]. LncRNAs are shown to take multiple roles in the different cellular mechanisms, including cellular proliferation, cell-cycle progression, apoptosis, motility, and cell survival [9]. Concerning the influence of lncRNAs in cancer development and progression, more than 20 polymorphisms in lncRNA genes are found to be associated with risks of several types of cancer [10]. LncRNAs are indicated to participate in the process of cancer development through various mechanisms [11]. Some lncRNA molecules act as oncogenes, whereas others act as tumor suppressors in a variety of cellular processes, including tumor cell proliferation, differentiation, invasion, and metastasis [11]. Due to the accessibility and availability of lncRNAs in the blood, saliva, and other body fluids of patients, these molecules could make potentially appropriate candidates for diagnosis and determining the course of cancer, especially OSCC as noninvasive biomarkers [8, 9, 12]. One of the most noticeable lncRNAs in this regard is lncRNA ANRIL [13].

LncRNA ANRIL, a long noncoding RNA in the INK4 locus, located in chromosome 9p21, is expressed at higher levels in several human cancer cells and thus has a crucial role in tumorigenesis [13, 14]. It has been shown that lncRNA ANRIL is overexpressed in OSCC, and plays an important role in the development and progression of OSCC; therefore, the gene associated with lncRNA ANRIL may be a potential risk factor for OSCC [12].

Studies have shown three major SNPs in lncRNA ANRIL associated with the risk of overall cancer risk. These SNPs include rs1333045 A/C [10, 15, 16], rs4977574 A/G [10, 15, 16], and rs10757278 A/G [10, 15, 16]. These SNPs were shown to be involved in breast cancer [15], esophageal SCC, and prostate cancer [16] of which, the rs4977574 A/G was found to play an imperative role in both breast and prostate cancer [15, 16]. These SNPs are demonstrated to hamper the function of lncRNA ANRIL by disrupting its splicing activity which would result in the modification of expression of genes modulated by lncRNA ANRIL including p15 (INK4b) leading to the likelihood of cancer development [17–19].

Despite the great significance of these SNPs, including the rs4977574, which is demonstrated to be one of the most influential SNPs in lncRNA ANRIL [10], no study has assessed the liability of rs4977574 A/G SNP of lncRNA ANRIL in the OSCC cases which, due to the current evidence, appears to be an attractive area of research in OSCC studies. Thus, this study aimed to evaluate the possible interdependence of rs4977574 of lncRNA ANRIL in patients with OSCC in both allelic form and resultant genotypes.

## 2. Materials and Methods

This case–control study was approved by the Ethics Committee of Shiraz University of Medical Sciences (IR.SUMS.DENTAL.REC.1400.147). In the current study, the participants of the study group were selected among the patients primarily diagnosed with OSCC who were admitted to the oral and maxillofacial surgery department of Rajaie Hospital and the Otorhinolaryngology Khalili Hospital. Those patients whose OSCC was approved by the full clinical and histopathological examination were included in our study, unless in case of a previous history of receiving cancer therapy, history of other malignancies, remote metastasis, genetic disorders, autoimmune diseases, or pregnancy. To compare the results of these patients with the normal population, healthy individuals, as the control group, were selected to match the age and gender of the patients with OSCC. The participants of the control group were randomly selected among the patients who referred to the Khalili and Rajaie hospitals with the condition of no history of cancer or autoimmune diseases among themselves or their first-degree relatives. A consent form was obtained from all the participants before the study.

In order to thoroughly understand the potential role of rs4977574 of lncRNA ANRIL in SCC, the relative frequency of the mutated allele was compared with the wild-type allele between the two groups. Moreover, the frequencies of the genotypes of the mentioned alleles were investigated in four types of inheritance models, including recessive, dominant, codominant, and overdominant models. The analysis of genotype frequencies based on each inheritance model was performed according to the following protocol:

  Recessive model: the major homozygote genotype + heterozygote genotype vs. the minor homozygote genotype
  Dominant model: the major homozygote genotype vs. heterozygote genotype + minor homozygote genotype
  Codominant model: the frequencies of each homozygote genotype vs. heterozygote genotype
  Overdominant model: the major homozygote genotype + minor homozygote genotypes vs. heterozygote genotype

Furthermore, in order to analyze the possible correlations between the SNP of rs4977574 of lncRNA ANRIL and the tumor characteristics, we assessed the correlation of the genotypes of this SNP with the tumor size (more or less than 4 cm), lymph node involvement (+: positive, −: negative), distant metastasis (+: positive, −: negative), TNM stage (1, 2, 3, and 4), tumor histological staging (poorly differentiated, moderately differentiated, and well-differentiated), and tumor site (tongue, buccal mucosa, lips, and other areas).

### 2.1. SNP Genotyping

Blood samples, obtained from the participants, were stored in an EDTA solution to prevent the clotting process. To extract DNA from the blood samples, the salting-out method was carried out. The purity of the final product was assessed by Eppendorf Biophotometer (Germany) with the ability to measure absorption rates at wavelengths ranging from 260 to 280 nm. The genomic sequence of lncRNA ANRIL was derived from the website of the National Institute of Biotechnology Information (NCBI). Specific regions of lncRNA ANRIL with SNPs were replicated by PCR technique and the final genotypes were determined using electrophoresis.

The locations of SNPs and the used primers, which were designed for T-ARMS-PCR as a simple means of pointing out the genotype of SNPs of lncRNA ANRIL (rs4977574), are shown in Table 1. The parts of lncRNA ANRIL (rs4977574) containing polymorphisms were duplicated with the PCR technique. Then, electrophoresis was conducted and the genotypes were determined. To carry out PCR, 20 *µ*L of a solution containing 50 ng of genomic DNA, 0.5 *μ*M of each primer, 1 U Taq of DNA polymerase, 250 *μ*M of dNTPs, and 1.5 mM of MgCl2 were used. The quantity and quality of DNA were determined by agarose gel 2% electrophoresis and UV spectrophotometry. The cyclic conditions of PCR included initial denaturation at 94°C for 5 min, followed by 30 cycles, annealing temperature for 45 s at 52°C, and extension temperature for 55 s at 72°C with a final extension of 72°C for 5 min. To validate the results, a percentage of samples was randomly selected for sequencing.

### 2.2. Statistics

To assess the age and gender distribution among the study and the control groups, an independent *T*-test and *χ*2 test were carried out, respectively. Furthermore, to compare alleles and genotype frequencies between the two groups, the *χ*2 test was applied. Moreover, to assess the possible correlation of the genotypes with the tumor characteristics, the *χ*2 test was applied. Odds ratio (OR) was scored to determine the strength of the association between ANRIL SNPs and the risk of OSCC. Besides, the difference between the observed genotypes' frequencies with the expected ones from the Hardy–Weinberg Equilibrium (HWE) was assessed using the *χ*2 test. The confidence interval (CI) of 95% was set for this study and the level of significance was 0.05. In all the statistical analyses in this study, SPSS software, version 16, was used.

## 3. Results

During this study, 106 patients with OSCC and 111 healthy individuals were included. The mean age of patients with OSCC was 61.59 ± 15.78, while the mean age for the healthy individuals was 51.38 ± 16.82. The statistical analysis of the age between the two groups was statistically insignificant (*p* value = 0.229). Moreover, the gender distribution among the groups was also reported as insignificant.  Table 2 represents the details of gender distributions among the study and the control groups.

To assess the possible covariations of the mutated allele G in rs4977574 in lncRNA ANRIL, the frequency of allele G was assessed between the study and the control group. The result indicated no statistically significant difference (*p* value = 0.701) (Table 3). The analysis of the genotype frequencies in each group showed no marked deviation from the HWE with *p* values of 0.843 in the study group, and 0.107 in the control group (Table 4).

Therefore, the appraisal of genotype analysis in patients with OSCC vs. healthy individuals was undertaken in four types of inheritance models (Table 5). The results indicated that in none of the inheritance models, a significant correlation between the genotypes and the existence of OSCC was discovered (*p* > 0.05).

Finally, a correlation test was performed between the genotypes and tumor size, tumor grade, tumor site, stage, and lymph node metastasis. The results indicated that the genotype GG was associated with tumor size smaller than 4 cm (*p* < 0.05); however, genotypes AA and AG did not show the same result (*p* > 0.05) (Tables 6 and 7). Concerning the level of OSCC histopathological differentiation, it was shown that the genotypes with at least one wild-type allele (A) were associated with the level of OSCC cells differentiation (both genotypes showed a higher prevalence among the samples with higher histological differentiation) (*p* < 0.05) (Tables 6 and 7). On the other hand, all the genotypes exhibited significant associations with the tumor site with most being reported in the tongue (*p* < 0.05) (Tables 6 and 7). Nevertheless, no statistically significant outcomes were found concerning the stage and the lymph node metastasis of the OSCC cells (*p* > 0.05) (Tables 7 and 8).

## 4. Discussion

The results of our study indicate no significant difference in the prevalence of the mutated allele G in the rs4977574 of lncRNA ANRIL between the normal population and patients with OSCC. Correspondingly, during the analysis of the frequency of genotypes of lncRNA ANRIL rs4977574 in all the inheritance models, no significant difference was discovered. Our findings are in line with the previous studies assessing the genotypes of lncRNA ANRIL rs4977574 in various types of cancers. In this regard, Khorshidi et al. [15] have appraised the mentioned SNP in patients with breast cancer. Similarly, they found no significant correlation between the genotypes in the study vs. the healthy individuals' group. In another study by Muraei-milan et al. [20], no statistical significance was reported in the correlation of genotypes of lncRNA ANRIL rs4977574 in papillary thyroid cancer in four types of inheritance models. Nevertheless, both studies have indicated the fact that when assessing a group of SNPs in lncRNA ANRIL as a haplotype, there is more likelihood to find correlations with cancer [15, 20]. Concerning the haplotype analysis, Muraei-milan et al. [20] have included lncRNA ANRIL rs4977574, as well as rs1333040 and rs10757274 in their haplotype analysis. In two of the haplotype models, in which the wild allele A of rs4977573 was involved, a significant correlation was found with papillary thyroid cancer. Furthermore, Khorshidi et al. [15] have included lncRNA ANRIL rs4977574 along with rs1333045, rs1333048, and rs10757278 in their haplotype analysis. Khorshidi et al. [15] merely reported one haplotype associated with the risk of breast cancer. These results suggest the potential role of rs4977574 in lncRNA ANRIL to take a role in cancer development and progression through its interaction with mutations in other sites of the DNA template of lncRNA ANRIL [15, 20].

Although the results of our study did not indicate any significant difference in the correlation of the mutation in rs4977574 of lncRNA ANRIL with OSCC, it is important to note that lncRNA ANRIL is suggested to have a significant role in cancer development. Several studies have reported the significance and the possible roles of lncRNA ANRIL in cancer development mechanisms. ANRIL is demonstrated to enhance in multiple cancers and diseases, including hepatocellular carcinoma [21], esophageal SCC [22], bladder cancer [23], prostate cancer [16], kidney malignancies [24], lung cancer [25], OSCC [12], and so forth [26]. This lncRNA seems to take part in cancer progression through various mechanisms, including epithelial–mesenchymal transition (EMT) [27], enhancement of cell proliferation [12], migration, and invasion, and eventually ceasing cell apoptosis [12]. The facilitation of the EMT process by lncRNA ANRIL is suggested to be activated by the ATM-E2F1 signaling pathway, which can increase the capability of the cancer cells to migrate and enhance their invasiveness [12, 28]. The positive impact of lncRNA ANRIL on the proliferation of cancer cells is suggested to be mediated via multiple mechanisms, including the phosphatidyl inositol 3-kinase/protein kinase B (PI3K/Akt) pathway [29], transforming growth factor-*β*/Suppressor of mothers against decapentaplegic (TGF- *β*/Smad) pathway [12], and suppression of p14 ARF [30], and p15INK4B [31]. Moreover, the knockdown of lncRNA ANRIL could result in cell cycle arrest in the G1 phase of the cell cycle [32]. The antiapoptotic effect of lncRNA ANRIL is shown to be mediated via TGF- *β*1 signaling pathway [12, 32]. Worth mentioning, some of the cell proteins that are highly involved in cell apoptosis, such as Bcl-2, caspase-3, and caspase-9 are affected by the level of expression of lncRNA ANRIL [32]. Therefore, lncRNA ANRIL seems to exert an imperative role in the cancer progression. Moreover, recent studies have focused on the mechanisms through which lncRNA ANRIL can enhance cancer cells resistance to chemotherapeutic medications. In this regard, Lee et al. [33], have indicated that the higher expression of lncRNA ANRIL is associated with higher drug resistance against Cisplatin and Doxorubicin in osteosarcoma cell lines. This was demonstrated through significant enhancement of half maximum inhibitory concentration (IC50) of the mentioned chemotherapeutic medications in the cell culture media [33]. Moreover, in a study by Wang et al. [34], it was shown that there is an imperative association between the gemcitabine-resistance in pancreatic cell lines and abnormal splicing of lncRNA ANRIL and methylation of N6-methyladenosine. Other studies in this regard, have also demonstrated the role of lncRNA ANRIL in resistance to chemotherapeutic medications, including the role of lncRNA ANRIL in cisplatin-resistance in retinoblastoma cells via the ATP-binding cassette transporter G2 (ABCG) expression pathway [35]. Furthermore, lncRNA ANRIL is reported to take role in bortezomib-resistance through epigenetic splicing of phosphatase and TENsin homolog deleted on Chromosome 10 (PTEN) [36].

Moreover, it should be noted that based on our analysis of the association of genotypes with the tumor size, tumor grade, lymph node invasion, and tumor stage, several findings indicative of the indirect role of lncRNA ANRIL rs4977574 mutation in OSCC were obtained. The authors of the current study found out that the genotype GG was associated with OSCC tumors smaller than 4 cm (which based on the TNM staging, is almost equal to T1 and T2 tumors smaller than 4 cm). Worth mentioning, genotypes AG and AA were significantly associated with the tumors histological differentiation (both genotypes exhibited higher frequencies in well-differentiated OSCC samples), whereas genotype GG did not show any marked association with the histological differentiation. Putting these results with the outcomes of the haplotype analysis of Muraei-milan et al. [20] indicates that the wild allele A seems to play a more active role in the enhancement of tumor size and tumor cell differentiation. Moreover, in the study by Muraei-milan et al. [20], it was found that in the only two haplotype models associated with the risk of papillary cancer development, only the wild-type allele A was present. Based on these outcomes, the authors of the current study suggest that the mutated allele G in the lncRNA ANRIL rs4977574 seems to have a negative role in cancer development during its interaction with other factors. Concerning the correlation of the assessed genotypes with the site of OSCC, it was found that all three genotypes (AA, AG, and GG) are associated with the occurrence of OSCC in the tongue, lip, and buccal mucosa, consecutively. Nevertheless, this significant association does not seem to be affected by the genotypes themselves since, based on the results of an epidemiological study on the OSCC patients in the same geographical region as we have obtained our samples [1], OSCC mostly occurs in tongue, followed by buccal mucosa, and maxillary gingiva. Regarding the impact of genotypes on the lymph node invasion or the stage of the OSCC, no significant results were found. This finding implies that even though the lncRNA ANRIL itself is found to be associated with lymph node metastasis and the TNM stage of cancer cells [37], the mutation of rs4977574 in lncRNA ANRIL does not substantially contribute to this process.

In light of the existing evidence, we can elucidate the significant influence of lncRNA ANRIL on the progression of OSCC cells. In this regard, Liu et al. [12] have stated that lncRNA ANRIL is significantly elevated in OSCC cells, yielding higher proliferation ability and suppression of apoptosis through the TGF- *β*/Smad signaling pathway. Furthermore, the outcomes of our study indicate the insignificant difference of mutation in the rs4977574 of lncRNA ANRIL, while exhibiting some interdependence between certain genotypes of the mentioned mutation with the smaller OSCC tumor size, and the level of differentiation in OSCC cells. From what has been gathered, we can conclude that despite the significant association of lncRNA ANRIL with OSCC, the mutation of lncRNA ANRIL rs4977574 does not directly influence this process. Nevertheless, the haplotype analysis and further evaluations on the possible interaction of lncRNA ANRIL with other tumorigenic factors would possibly broaden innovative perspectives on this process. Moreover, due to the incompleteness of data concerning the tumor characteristics of some of patients, although our statistical analyses have indicated novel findings in this regard, the interpretation of our results should be performed with caution. We also recommended further investigations to clarify these possible associations in this regard. Worth mentioning, further studies are encouraged with multiple ethnical groups to assess the influence of ethnical variations on this genetic association [38].

## 5. Conclusion

In the light of the current findings, we can state that the mutation of lncRNA ANRIL rs4977574 A/G does not result in any significant association with the presence of OSCC at both allelic and genotypic levels. However, the correlation of specific genotypes with tumor size, OSCC cell differentiation, and the tumor site could indicate its possible indirect role in the pathophysiology of OSCC. On the other hand, the other tumor characteristics, including tumor grade and lymph node metastasis did not exhibit any significant correlation.

## Figures and Tables

**Table 1:** Presentation of the primers used for the detection of rs4977574 polymorphism in the lncRNA ANRIL gene.

SNP	Product size	Chromosome position	Primers	Sequence (5ʹ to 3ʹ)
rs4977574 lncRNA ANRIL	Outer primers: 335 bp	9P21.3 22098575	FO	CACCATTCTTTCTGAAACAACAGGATAT
			RO	AAGGCTCTGACATTTCTAACTCTCTGA
	G allele: 226 bp		FI	TTGAGGGTACATCAAAAGCATTCTATATCG
	An allele: 166 bp		RI	TTTATTAGAGTGACTTGAACATCCCGT

**Table 2:** Illustration of gender distribution among the group of patients with OSCC and healthy individuals.

Characteristics	Study group (%)	Control group (%)
Number	106	111
Male	54 (50.94%)	53 (47.74%)
Female	52 (49.05%)	58 (52.25%)
*p* Value	0.637	

**Table 3:** Assessment of the frequency of mutated allele G in rs4977574 of lncRNA ANRIL in patients with OSCC vs. the healthy individuals.

rs4977574	Allele	Study group	Control group	OR	*p* Value
	A	104	113	–	–
	G	108	109	1.08 (0.74–1.57)	0.701

*Note*: OR: odds ratio.

**Table 4:** Representation of the frequency of genotypes of rs4977574 of lncRNA ANRIL in patients with OSCC and the healthy individuals and their deviation from the HWE.

rs4977574 genotypes	AA (%)	AG (%)	GG (%)	*p* Value of HWE
Study group	25 (23.58%)	54 (50.94%)	27 (25.47%)	0.843
Control group	33 (29.72%)	47 (42.34%)	31 (27.92%)	0.107

*Note*: HWE: Hardy–Weinberg equilibrium.

**Table 5:** Evaluation of the genotypes of rs4977574 of lncRNA ANRIL in patients with OSCC vs. the healthy individuals in different inheritance models.

SNP	Genotype	Inheritance model	Study group	Control group	OR	*p* Value
rs4977574	AA	Codominant	25	33	–	–
	AG		54	47	1.52 (0.79–2.91)	0.209
	GG		27	31	1.15 (0.55–2.39)	0.708
	AA + AG	Recessive	79	80	–	–
	GG		27	31	0.88 (0.48–1.61)	0.682
	AA + GG	Overdominant	52	64	–	–
	AG		54	47	1.41 (0.82–2.41)	0.204
	AA	Dominant	25	33	–	–
	AG + GG		81	78	1.37 (0.748–2.511)	0.307

*Note*: OR: odds ratio, SNP: single nucleotide polymorphism.

**Table 6:** Representation of the prevalence of each genotype in different tumor sizes, tumor grades (histopathological differentiation), and tumor sites.

Genotypes	Tumor size		Tumor Grade (histopathological differentiation)		Tumor site			
	≥4 cm	<4 cm	Well-differentiated	Moderately differentiated	Tongue	Buccal mucosa	Lip	Other areas
AA	29.31%	70.68%	66.08%	33.91%	75%	3.44%	1.72%	19.82%
AG	33.62%	66.37%	71.30%	28.69%	79.82%	1.75%	1.75%	16.66%
GG	25%	75%	53.91%	46.08%	55.1%	3.44%	1.72%	39.65%

**Table 7:** Representation of the estimated *p*-values concerning the association of each genotype with tumor size, tumor grade, tumor site, tumor stage, and lymph node metastasis.

rs4977574 Genotypes	Tumor size^1^	Tumor grading^2^	Tumor site^3^	Tumor stage^4^	Lymph node metastasis^5^
AA	0.090	0.041^*∗*^ (Well-differentiated)	0.007^*∗*^ (Prevalent in tongue)	0.273	0.617
AG	0.068	0.011^*∗*^ (Well-differentiated)	<0.001^*∗*^ (Prevalent in tongue)	0.455	0.857
GG	0.025^*∗*^ (Less than 4 cm)	0.782	0.005^*∗*^ (Prevalent in tongue)	0.172	0.819

*Note*:  ^*∗*^Statistically significant, ^1^tumor size (more or less than 4 cm), ^2^lymph node involvement (+: positive, −: negative), ^4^TNM stage (1, 2, 3, and 4), ^5^tumor histological staging (poorly differentiated, moderately differentiated, well-differentiated), ^6^tumor site (tongue, buccal mucosa, lips, and other areas).

**Table 8:** Representation of the prevalence of each genotype in different tumor stages, and lymph node metastasis cases.

Genotypes	Tumor stage				Lymph node metastasis	
	1	2	3	4	+	−
AA	9.53%	50.43%	40%	0%	56.52%	43.47%
AG	11.40%	30.70%	27.19%	30.70%	51.30%	48.69%
GG	18.42%	31.57%	43.85%	6.14%	53.04%	46.95%

## Data Availability

The data used in this study are available on a reasonable request from the corresponding author.
